# Transforming static interfaces into tactile channels with steerable transdermal foci

**DOI:** 10.1126/sciadv.aef2372

**Published:** 2026-07-08

**Authors:** Qiutong Liu, Yi Tang, Jingyue Luo, Wenhao Xue, Ziyi Lin, Zhirui Liu, Miao Luo, Jing Wang, Xuezhi Ma, Jiezhou Pan, Yuan Ma

**Affiliations:** ^1^ CTSIRI-PolyU Joint Lab, Department of Mechanical Engineering, The Hong Kong Polytechnic University, Hong Kong SAR, China.; ^2^ Research Institute for Intelligent Wearable Systems, The Hong Kong Polytechnic University, Hong Kong S.A.R., China.; ^3^ Sanming Medical and Polytechnic Vocational College, Sanming, China.; ^4^ Department of Respiratory Medicine, The First Affiliated Hospital, Army Medical University, Chongqing, China.; ^5^ Department of Mechanical Engineering and State Key Laboratory of Mechanical System and Vibration, Shanghai Jiao Tong University, Shanghai, China.; ^6^ Quantum Innovation Centre (Q.InC), Agency for Science, Technology and Research, 4 Fusionopolis Way, Kinesis #05, Singapore, Singapore.; ^7^ The Research Institute for Artificial Intelligence of Things (RIAIoT), The Hong Kong Polytechnic University, Hong Kong S.A.R., China.; ^8^ PolyU-Daya Bay Technology and Innovation Research Institute, Huizhou, China.

## Abstract

Grasping objects such as handles, mice, and phones plays a central role in human-machine interaction, yet the contact surfaces of the hand, despite their exquisite tactile sensitivity, are rarely exploited as channels for information transfer. Here, we introduce transdermal all-directional targeting (TADT), a static-contact interface design strategy that converts passive grip surfaces into active tactile displays, allowing users to grasp stably while receiving spatially and temporally evolving information on the fingertips. TADT enhances sub-actuator-pitch energy delivery by integrating a microtextured skin-coupling interface with phase-controlled vibrotactile actuation, thereby generating steerable three-dimensional tactile foci beneath the skin, enabling >200-hertz vibration and sub-actuator-pitch spatial super-resolution. Psychophysical experiments show that TADT lowers detection thresholds by 30% and reduces power consumption by 80% compared with designs without TADT. Using static grip alone, users reliably identified six directional cues and completed nonvisual indoor navigation and virtual reality interaction tasks, demonstrating a compact, energy-efficient modality for expressive tactile communication.

## Teaser

Subdermal vibrotactile focusing under static contact delivers steerable programmable 3D touch cues for human-machine interaction.

## INTRODUCTION

Grasping everyday objects such as handles, mice, and cellphones is fundamental to human-machine interaction (*1*–*3*). While this static contact is essential for maintaining grip, it limits the ability of hands to perceive tactile information despite their high sensitivity (*4*–*6*). To compensate, current systems increasingly rely on supplementary interfaces such as visual displays, auditory cues, and wearable patches to facilitate communication between users and machines (*7*–*15*). These additional channels can elevate cognitive load, disrupt natural interaction, and increase energy consumption. Harnessing the untapped potential of static contact surfaces offers a promising pathway to more intuitive and efficient human-machine interfaces (*16*–*19*).

However, existing ways to deliver information through these interfaces typically rely on simple actuators such as linear resonant actuators, eccentric rotating mass motors, or thermal regulators that provide only single-point or low-resolution information (*20*–*23*). The limited richness of tactile cues in these static interfaces arises not only from actuator constraints such as sizes and actuation frequencies but also from poor energy coupling at the skin interface. The outermost stratum corneum absorbs and attenuates vibrations due to its high rigidity, hindering effective stimulation of deeper mechanoreceptors that mediate nuanced tactile perception (*24*–*31*). Consequently, grasp-based contact regions such as fingertips, metacarpal areas, and hypothenar areas of the hands have been left almost untapped as meaningful communication channels.

**Fig. 1.**
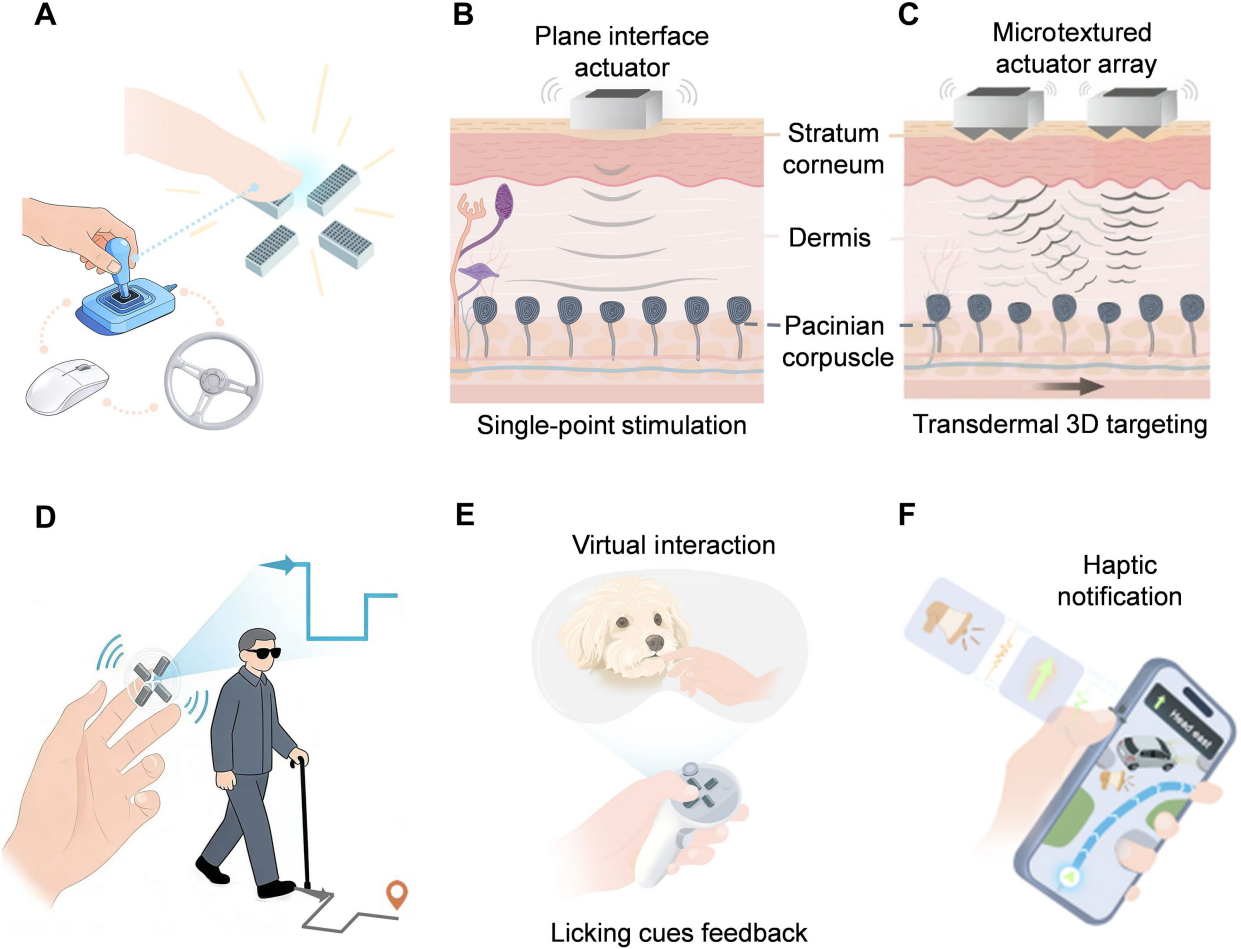
Concept of TADT and its applications. (**A**) Static hand-device contact surfaces as untapped channels for tactile communication. (**B**) Schematic of conventional vibration confined to the skin surface with plane actuator interfaces. (**C**) Schematic of deep-layer vibration transmission enabled by microtextured integrated actuator interfaces. (**D**) Tactile navigation via steerable transdermal foci for vision-impaired users. (**E**) Realistic texture rendering and interaction in virtual or augmented reality environments. (**F**) Directional alerts and guidance assistance systems through spatially controlled stimulation.

Here, we introduce transdermal all-directional targeting (TADT), a design strategy of static-contact interface that transforms previously passive grasping regions into rich channels for programmable tactile feedback (Fig. 1A). This strategy complements several recent advances in wearable haptic and electromechanical interfaces. For example, recent work has introduced a pneumatic haptic platform that combines multiscale actuator design with soft, body-conformal integration, highlighting the advantages of pneumatic actuation for large-area feedback and wearable adaptability (*32*). Other studies have demonstrated flexible piezoelectric architectures with excellent mechanical compliance and strong potential for seamless body integration, illustrating the value of soft electromechanical systems for wearable human-machine interfaces (*33*). In addition, related efforts have shown how soft electromechanical platforms can integrate remote actuation, sensing, and multifunctionality within compact compliant structures (*34*). TADT addresses a complementary design space: Rather than emphasizing large-area pneumatic rendering or flexible body-mounted electromechanical sensing, TADT focuses on compact static-contact surfaces and uses microtextured mechanical coupling together with phase-controlled vibrotactile actuation to enable steerable subdermal tactile stimulation.

TADT integrates phase-controlled vibrotactile focusing with a microtextured surface to overcome stratum corneum impedance, delivering spatially steerable, three-dimensional (3D) tactile cues deep into the skin tissue with sub-actuator-pitch spatial super-resolution using vibrotactile actuators (Fig. 1, B and C). Here, “spatial super-resolution” refers to the generation of multiple virtual focal positions within the spacing between physically separated actuators, rather than to the physical pitch of the actuator hardware itself. This approach not only activates deep mechanoreceptors such as Pacinian corpuscles but also achieves marked energy efficiency, reducing power demand by up to 80% compared with vibrotactile designs without TADT. We demonstrate the capabilities of the system through representative scenarios, including real-world navigation tasks in which users follow vibrotactile cues to move and act without visual guidance (Fig. 1D). We further show affective cues in virtual reality, such as simulating the direction of a dog licking the hand (Fig. 1E). By reclaiming underused static contact areas and enabling volumetric tactile stimulation without additional sensory load, TADT establishes a foundation for multitasking, assistive communication, and immersive XR applications.

## RESULTS

### Enhancing skin vibration perception with microtextured surfaces

The TADT strategy enhances sub-actuator-pitch energy delivery by integrating sharp microscale textures onto the surfaces of vibrotactile actuators. In the present design, these textures are cone-shaped protrusions with an approximately conical geometry and sharp apexes (fig. S1). The representative structure used in this work has a height of 200 μm relative to the base plane, a base diameter of 200 μm, and a center-to-center spacing of 400 μm. These textures are engineered with heights comparable to the thickness of the stratum corneum, allowing them to gently indent this outer layer without penetrating or damaging viable skin tissue (Fig. 1C). This controlled indentation improves mechanical coupling and substantially reduces the attenuation typically imposed by the stratum corneum. We further compared several representative geometries in simulation, including a sharp cone, a rounded-corner protrusion, and a rectangular protrusion (fig. S7). All three shapes enhanced subsurface coupling to a similar extent, while the rectangular protrusion showed a slightly higher response under idealized simulation conditions, likely because its larger effective contact area and more stable local indentation produced slightly stronger coupling into the tissue. When multiple microtextured actuators operate as a small array, phase modulation across elements produces virtual focal points with spatial super-resolution. The design of a microtextured surface is necessary to deliver enough energy into the tissue for further phase manipulation.

The biological mechanism of this enhancement is that the stratum corneum forms a stiff outer barrier that attenuates surface vibration, whereas Pacinian corpuscles are located deep in the dermis and are specialized for high-frequency vibration near the operating range used in this work (*30*). Prior studies suggest that the layered structure and depth of the Pacinian corpuscle shape how mechanical deformation is transmitted to the afferent terminal (*32*), and recent work further indicates that lamellar cells within these corpuscles actively contribute to touch detection (*35*). In this context, we interpret the effect of the microtextured interface as improved mechanical coupling at the skin boundary, which increases local deformation transfer through the outer skin layers and enhances the vibration reaching deeper mechanoreceptive regions, thereby strengthening tactile perception.

**Fig. 2.**
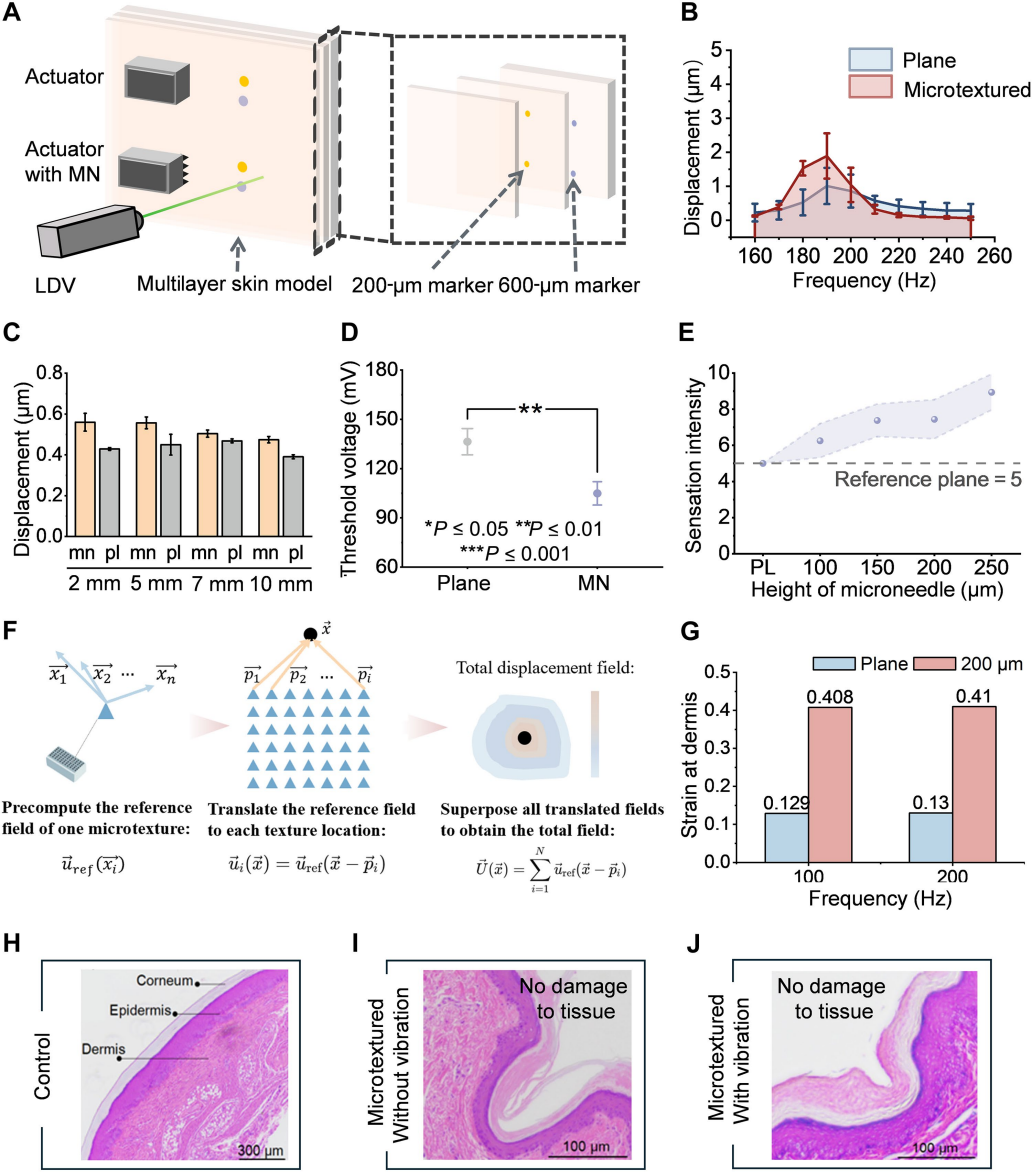
Enhanced deep-tissue coupling and perception via microtextured integrated vibrotactile interfaces. (**A**) Schematic of LDV measurement setup using multilayer PDMS with embedded depth markers. (**B**) Displacement amplitudes at 600-μm depths during *Y*-axis vibration for actuators with and without microtexture. (**C**) Lateral vibration propagation across increasing horizontal distances at 600-μm depth for microtextured (mn) and plane (pl) interfaces. (**D**) Comparison of human perceptual threshold voltages between microtextured (MN) and plane actuator interfaces. (**E**) Subjective intensity ratings for different microtexture heights relative to the plane interface baseline. (**F**) Schematic illustration of the wave-field superposition algorithm. A reference displacement field is first computed for a single microtexture. This reference field is then translated to the location of each texture in the array, and the contributions from all textures are linearly superposed to obtain the total displacement field of the microtexture array. (**G**) Simulated strain at the dermis across different frequencies for plane and microtextured interfaces with varying needle heights. (**H**) Representative hematoxylin and eosin (H&E) histology image of rat palm skin after contact with a plane actuator, showing intact skin layers. (**I**) Representative H&E histology image of rat palm skin after static contact. (**J**) Representative H&E histology image of rat palm skin after 15 min interaction with a microtextured actuator, showing surface deformation without tissue damage. Complete histology images from all tested sites are provided in fig. S10. In both (H) and (J), the microtextures indented the superficial skin surface but did not pierce through the epidermis, indicating that neither static contact nor vibration caused structural skin injury under the tested conditions.

To explore how microtextured surfaces with sharp features can improve vibrotactile energy delivery for enhanced vibration perception, we created a three-layer physical skin model that mimics the mechanical properties of human skin (Fig. 2A). The stratum corneum and epidermis were simulated using optically transparent polydimethylsiloxane (PDMS) layers with tailored Young’s moduli corresponding to respective skin layers. Meanwhile, the dermis was modeled using a silicone-based material with a comparable modulus (*36*–*39*). The transparency of the upper layers enabled laser Doppler vibrometer (LDV) measurements through the skin model, allowing noninvasive observation of vibration distributions within the dermal-equivalent layer. Microscale markers were embedded within the transparent skin model at depths of 200 μm (*30*, *40*, *41*) and 600 μm (*32*, *42*) to serve as fixed measurement points for depth-resolved LDV analysis of vibration within the multilayer structure.

We compared stimulation effectiveness between a plane and a microtextured integrated actuator (Fig. 2B). The scanning electron microscopy images of the specific morphology of the microtextured interface are shown in fig. S1. At a depth of 200 μm, both showed similar displacement, especially near 180 to 200 Hz (*40*, *43*–*46*) (see fig. S2 for details). However, at 600 μm, the microtextured interface retained higher amplitude, indicating better coupling into deeper layers by reducing energy loss across skin boundaries. We measured displacement at 2- to 10-mm distances from the actuator at 600-μm depth to assess lateral propagation (Fig. 2C). The microtextured interface (denoted as mn in the figure, reflecting its microneedle-inspired surface geometry) consistently maintained stronger signals across all distances, indicating improved long-range lateral transmission through the dermis-mimicking layer where the Pacinian corpuscle is located. These findings demonstrate that microtextured surfaces enhance vertical and lateral vibratory transmission, supporting their use in spatially extended haptic feedback. Their effect is especially pronounced in preserving vibrational energy over distance at 600-μm depth, which is critical for applications requiring distributed mechanical stimulation or sensing across a larger tissue area.

Subjective evaluation further confirmed the effectiveness of perceptual effects. Using a three-phase adaptive staircase method, we measured the sensory threshold (ST) (*43*, *44*), with driven voltage required to detect the vibratile cue recorded across interface types (see Materials and Methods for full protocol). As shown in Fig. 2D, the microtextured interface reduced the average detection threshold by ~30% compared with the plane interface (*P* < 0.01, repeated-measures analysis of variance). This reduction in voltage demand translated into a ~44% decrease in electrical power consumption, consistent with the improved transmission efficiency provided by the microtextured surface.

We further evaluated the perceptual effect of interface geometry using a relative subjective intensity-rating experiment with microtextured surfaces of different heights (100 to 250 μm) under identical excitation frequency and amplitude. In each trial, participants first touched the planar actuator interface, which was defined as the reference and assigned a fixed score of 5, and then touched the microtextured actuator under the same driving condition. Participants rated the perceived intensity of the microtextured interface relative to this reference: Scores above 5 indicated stronger perceived vibration than the planar interface, a score of 5 indicated comparable intensity, and scores below 5 indicated weaker perceived vibration. As shown in Fig. 2E, even the shortest microtexture was rated above the planar reference, and the perceived intensity increased approximately linearly with microtexture height, confirming that increased surface protrusion enhances tactile perception.

To further assess whether this perceptual enhancement remains robust under practical user-dependent conditions of skin hydration, normal contact force, and stratum corneum thickness, we conducted additional subjective comparisons using the plane interface as a reference (assigned intensity of 5) and invited participants to rate the perceived intensity of the microtextured interface under matched driving conditions. For fingertip moisture, participants’ fingertips are wiped with deionized water, alcohol, or artificial sweat to simulate wet, dry, and sweaty conditions. As shown in fig. S3, the microtextured interface was consistently rated above the plane reference in all three conditions, indicating that the perceptual benefit was preserved despite variations in surface moisture. We next evaluated the effect of contact pressure, with participants adjusting their fingertip force based on the pressure sensor readout to achieve normal loads of 4 and 8 N, which lie within the typical range experienced during everyday object contact and grasping. As shown in fig. S4, the perceived intensity of the microtextured interface remained higher than that of the plane interface at both pressure levels. A moderate decrease in rating was observed at 8 N relative to 4 N, suggesting that larger preload may partially reduce the relative perceptual gain while not eliminating the enhancement effect.

Last, to examine whether interindividual differences in skin properties influence perception, we grouped participants into male and female cohorts as a practical, coarse proxy for variation in fingertip skin morphology. This grouping was motivated by prior reports that epidermal and stratum corneum thickness vary with body site, age, and sex and that fingertip stratum corneum thickness is thinner in women than in men (*40*). As shown in fig. S5, both groups consistently rated the microtextured interface above the plane reference, with only a modest difference in mean intensity between groups. These results indicate that the perceptual enhancement produced by the microtextured interface is not restricted to a single skin condition but remains observable across variations in moisture state, contact pressure, and participant group. The corresponding experimental details have been included in the “Subjective robustness evaluation” section.

To further examine the effective bandwidth of this perceptual enhancement, we also compared subjective perception of vibrotactile actuation with microtextured interface at driving frequency of 50 and 100 Hz. As shown in fig. S6, the microtextured interface produced a clear enhancement at 100 Hz, with ratings generally above the plane reference. In contrast, at 50 Hz, the perceived intensity of the microtextured interface was lower than that of the plane interface. These results indicate that the perceptual benefit of microtextured interface is frequency-dependent rather than uniform across the vibrotactile spectrum, with stronger enhancement emerging toward the higher-frequency range. This trend is consistent with the design rationale of TADT, which improves energy delivery to deeper tissue layers and is therefore better matched to vibration channels associated with deeper mechanoreceptors.

### Computational analysis of microneedle array interfaces on multilayer skin

To quantitatively understand how microtextured arrays influence the transmission of mechanical vibrations in skin-like tissue, we developed a computational framework called the microtextured array simulation algorithm. The framework uses a linear superposition strategy based on the translational invariance of the medium and the assumption of linear wave propagation (Fig. 2F). Detailed implementation and model configuration are described in Materials and Methods. The model was implemented using COMSOL Multiphysics and represents a 3D multilayer structure composed of stratum corneum, epidermis, and dermis. Each layer incorporates specific mechanical properties, including thickness, Young’s modulus, and Poisson’s ratio calibrated to approximate human skin characteristics (*47*–*49*). A detailed schematic of the model configuration is provided in Materials and Methods.

We further used the computational model to compare dermal strain transfer at two representative excitation frequencies, 100 and 200 Hz, for the plane and microtextured interfaces. As shown in Fig. 2G, the microtextured interface consistently produced substantially higher strain in the dermis layer than the plane interface at both frequencies. The simulated dermal strain for the 200-μm microtextured interface remained nearly unchanged between 100 and 200 Hz, and the same trend was observed for the plane interface. Notably, the 200-μm microtexture consistently generated the highest strain amplitudes at all tested frequencies, which corresponds approximately to the upper bound of epidermis thickness in human skin (*50*) and thus maximizes coupling while avoiding penetration into viable epidermal layers. This weak frequency dependence mainly reflects the simplifying assumptions of the present model, in which the skin layers were treated as frequency-independent elastic materials rather than viscoelastic media. We adopted this formulation to isolate the role of interface geometry in dermal strain transfer and to provide a first-order comparative assessment of microtextured versus plane coupling under matched constitutive assumptions. Such elasticity-based simplifications have also been used in prior tactile biomechanics studies as tractable frameworks for analyzing mechanical transmission and tactile discrimination (*47*, *51*). Recent reviews of tactile simulation models likewise note that simplifying assumptions are commonly adopted to make skin-mechanoreceptor modeling computationally tractable. Therefore, Fig. 2G is intended to highlight the geometry-dependent enhancement of dermal strain transfer by the microtextured interface, rather than to fully reproduce the frequency-dependent viscoelastic behavior of biological skin. We further analyzed subsurface vibration behavior by observing time-domain displacements at a fixed point 7 mm laterally and 600 μm deep to confirm that our microtextured interface enhances deep coupling compared with a plane patch (see fig. S3 for details). We also simulated different microtextured array layouts, square, circular, and sector, to uncover geometry-dependent trends, offering actionable insights for optimizing array design (fig. S4).

We evaluated the potential invasiveness of our 200-μm microtextured interface using Sprague-Dawley rats as the animal model, focusing on the plantar surfaces of the forepaws and hindpaws, which were selected for their consistent skin thickness and frequent use in tactile studies (*52*, *53*). Static contact and 15-min vibratory stimulation were applied at three sites per paw. Hematoxylin and eosin (H&E) (*54*) staining revealed only superficial indentation of the stratum corneum without penetration or disruption of the viable epidermis (Fig. 2, H to J). Specifically, Fig. 2H shows the control condition with a plane actuator interface, Fig. 2I shows static contact with the microtextured interface, and Fig. 2J shows the skin after 15 min of vibratory interaction with the microtextured interface. In Fig. 2 (I and J), the microtextures indented the superficial skin surface but did not pierce through the epidermis, indicating that neither static contact nor vibration caused structural skin injury under the tested conditions. The dermal architecture, collagen structure, and absence of inflammatory infiltration remained intact across all conditions (see fig. S5 for details). These results confirm that our microtextured interface provides localized, noninvasive deformation while preserving skin integrity, supporting its safety and potential for long-term human use.

### Generating dynamic focus with microtextured actuator array

**Fig. 3.**
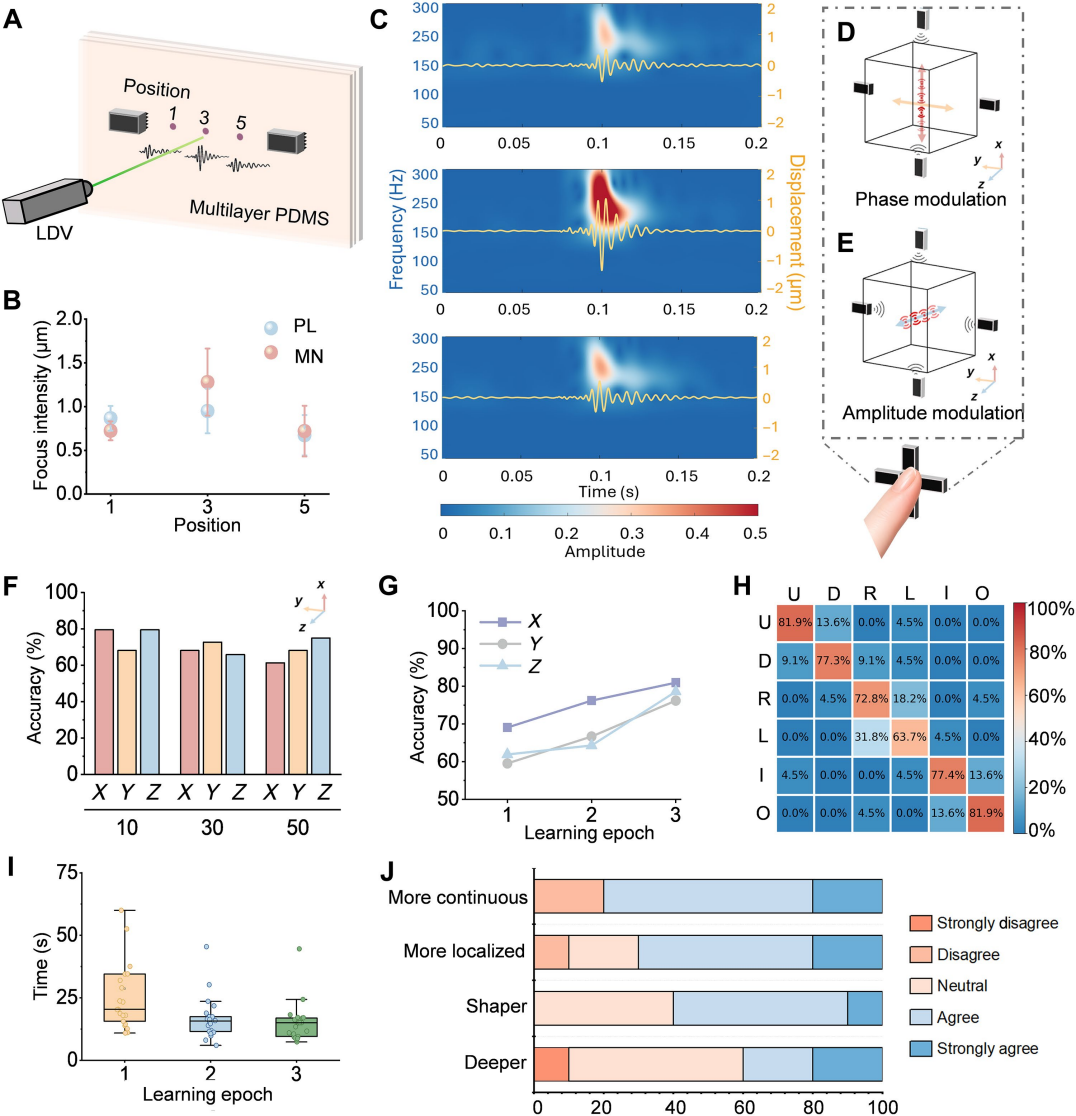
Spatial focusing and dynamic three-axis control of vibrational stimulation using microtextured integrated actuators. (**A**) Experimental setup for spatial vibration focusing using dual actuators on a multilayer PDMS model. (**B**) Measured focal intensities at different positions for plane and microtextured interfaces. (**C**) Continuous wavelet transform of vibration signals recorded at five spatial positions, highlighting energy concentration at the illusory vibration. (**D**) Schematic and driving signals for lateral (*XY* plane) focus steering via phase modulation between actuators. (**E**) Schematic and driving signals for depth-wise (*Z* axis) focus modulation via amplitude adjustment between actuators. (**F**) Recognition accuracy as a function of focal-path density for the *X*, *Y*, and *Z* axes. The three colors correspond to directional trajectories rendered with 10, 30, and 50 focal positions, respectively. (**G**) Learning curves showing recognition accuracy improvements over successive training epochs. (**H**) Confusion matrix of direction recognition accuracy across six dynamic targeting movement directions, upward (U), downward (D), rightward (R), leftward (L), inward (I), and outward (O). (**I**) Response time reductions across learning epochs during directional tactile recognition tasks. (**J**) Qualitative comparison of focused subdermal feedback and conventional surface vibration using a five-point Likert scale.

Building on enhanced subsurface vibration transmission, we further explored the generation of dynamic focus within a multilayer skin-mimicking PDMS model. As depicted in Fig. 3A, two actuators (either microtextured integrated or plane) were mounted on opposite sides of the model, with phase- and amplitude-controlled signals creating constructive interference at the internal focal point (position 3). Lateral displacement measurements at three positions (1, 3, and 5) showed that both setups produced maximal response at position 3. We compared focal displacement amplitudes between planar and microtextured coupling interfaces. Under identical drive conditions, the microtextured interface achieved a focal displacement of ~1.3 μm compared to ~0.8 μm for the plane interface (Fig. 3B). Assuming linear proportionality between drive voltage and displacement in the small-signal regime of the actuator, this corresponds to a voltage requirement reduced to ~62% of the planar case. Because electrical power scales quadratically with voltage, the estimated power consumption for generating the same target displacement is reduced to ~38% (~62% saving). This effect can be considered in conjunction with the independently measured reduction in ST, where the required voltage decreased by ~30% (from 139 mV to 104 mV), corresponding to a ~44% reduction in electrical power. The synergistic combination of microtextured coupling and phase-focused actuation suggests improved mechanical efficiency and that the overall drive power requirement could be reduced to less than 20% of the planar baseline (~80% power saving). Time-frequency waveforms analysis (*55*) using continuous wavelet transform further revealed a temporally confined spectral burst around 200 Hz exclusively at the focus (Fig. 3C), providing evidence that microtextured interfaces enable deep-layer, spatially precise vibrotactile focusing. Additional measurements of the in-plane vibration signals at lateral positions 1, 3, and 5 within the 600-μm depth region further confirmed that the focused vibration remained spatially localized at depth, with the strongest response observed at the focal center (fig. S11).

We implemented a dynamic three-axis tactile focusing system using four actuators arranged in two orthogonal pairs. Phase modulation and amplitude modulation play different roles in the present system. Phase modulation is used primarily for lateral steering of the focal stimulus in the *XY* plane. When opposing actuators are driven at the same frequency but with different phase offsets, the position of constructive interference shifts between them, leading to a lateral translation of the perceived focal point across the fingertip surface (Fig. 3D). By contrast, we use amplitude modulation to evoke *Z*-direction perception, with changes in the relative amplitude between actuator pairs altering the strength and focus of the central stimulation and thereby creating a sensation of inward or outward motion on the skin. Depth-wise modulation (*Z* axis) was controlled by varying the amplitude ratio (Fig. 3E) between actuator pairs, thereby generating an illusion that the focal point is shifting inward (toward the dermis) or outward (toward the surface). The excitation signals are shown in figs. S6 and S7. A dynamic visualization of the four-actuator arrangement and the focal evolution under phase and amplitude modulation is provided in movie S3. While prior studies have reported phantom or funneling tactile illusions generated by interpolating intensity or phase across spatially separated actuators to evoke a perceived moving focus on the skin surface, these effects have largely remained confined to lateral, surface-level cues. For example, inverse filtering has been used to improve spatial precision across sparse actuator arrays (*56*), and intensity interpolation has been shown to induce apparent motion between discrete contact points. However, such approaches are fundamentally constrained by skin mechanics and lack control over depth. In contrast, our system achieves volumetric control of the perceived focal location within skin-like tissue by combining phase-coherent wave interference with microtextured coupling, enabling controlled steering of tactile foci not only laterally but also along the depth axis. This enables subdermal steering of vibrotactile stimuli through static contact, without requiring finger movement or mechanical translation.

Here, the reported spatial super-resolution refers to virtual focal positions rendered within the spacing between physically separated actuators, rather than to the physical pitch of the actuator hardware itself. In the current device, opposing actuators along each stimulation axis were separated by 1 cm center to center, and phase-controlled excitation enabled the rendering of 10, 30, and 50 virtual focal positions within this interval, corresponding to nominal focal step sizes of ~1.0, 0.33, and 0.20 mm, respectively. The number of actuators that can be placed on the fingertip is constrained mainly by actuator size and available contact area of the fingertip. In the current implementation, each actuator has a lateral size of ~5 mm, such that up to eight actuators could in principle be arranged around the fingertip, whereas the present study used four actuators. Within this framework, *X*- and *Y*-direction cues are generated by lateral steering of the focal vibration position across the fingertip surface, whereas the *Z*-direction cue is generated by modulation of the central focal intensity to induce an inward/outward depth percept rather than fixed point-by-point depth addressing.

We conducted subjective experiments to evaluate the effectiveness of the rendered tactile cues in conveying directional information by six-axis discrimination tasks (see Materials and Methods for full protocol). We compared recognition accuracy across different numbers of targeting points per direction, namely, 10, 30, and 50, to determine the optimal path resolution for perceptual discrimination. As shown in Fig. 3F, the highest recognition performance was achieved with ~10 targeting points per path along the *X* and *Z* axes. In comparison, 30 targeting points yielded optimal accuracy for the *Y* axis. Both lower and higher densities led to reduced recognition accuracy, potentially due to insufficient spatial sampling in the former and perceptual saturation in the latter.

Through a longitudinal study, we further evaluated how users adapted to the system over time. Participants performed the directional recognition task across three learning epochs: without prior exposure (epoch 1), after 30 min of feedback-informed practice (epoch 2), and after an additional 30 min of feedback-informed practice (epoch 3), during which the correct direction was revealed after each trial. As shown in Fig. 3G, recognition accuracy improved progressively across all axes (*X*, *Y*, *Z*), with the *X* axis consistently yielding the highest accuracy. Figure 3H demonstrates that participants achieved high overall recognition rates, with average accuracy exceeding 80% across all directions. Vertical motions (upward and downward) were identified more reliably than lateral or depth-wise cues, revealing axis-dependent differences in tactile discriminability. Correspondingly, the response time required to complete the six-direction recognition task decreased across learning epochs Fig. 3I, indicating increasing familiarity and confidence in interpreting the focal cues.

We further tested whether TADT remains effective when the actuators are arranged on noncoplanar surfaces. As shown in fig. S14, directional recognition accuracy remained high for both convex and concave actuator layouts, reaching 90 and 80%, respectively. This result suggests that the proposed strategy is not restricted to planar arrangements, although surface curvature influences the consistency of mechanical coupling and thus perceptual performance.

We further conducted a qualitative subjective evaluation to characterize how the focused subdermal feedback was perceived relative to conventional surface vibration. Participants compared the dynamic focal feedback generated by our device with the surface vibration produced by a traditional single actuator and rated four perceptual descriptors using a five-point agreement scale: more continuous, more localized, sharper, and deeper. Here, a score of 1 indicated strong disagreement, and a score of 5 indicated strong agreement with the statement that the focused feedback exhibited the corresponding sensation more strongly than the conventional surface vibration. As shown in Fig. 3J, participants generally described the focused feedback as more continuous, more localized, sharper, and deeper. Agreement was strongest for the descriptors “more localized” and “more continuous,” whereas “sharper” and “deeper” also received predominantly positive ratings. These results indicate that the proposed focused stimulation is perceived not simply as stronger vibration, but as a qualitatively distinct tactile experience with enhanced spatial concentration and an apparent subsurface character.

### Static-contact tactile guidance for accessible path tracing and immersive VR

We designed an indoor mobility scenario in which participants moved through a classroom while relying on tactile cues. Wearing an eye mask and noise-canceling earmuffs, participants received dynamic focal signals on the fingertip that indicated forward, backward, left, and right movements, whereas inward-outward shifts conveyed “raise hand” and “lower hand” commands needed to retrieve a notebook placed on a desk. After a brief familiarization period, participants successfully followed the tactile instructions to traverse the room, adjust hand height, and locate the target object without any visual or auditory input (movie S1). This demonstration shows that users can intuitively interpret multidimensional cues rendered through static contact, enabling nonvisual, hands-busy navigation and object-interaction tasks. Such capability suggests potential applications in assistive navigation, training environments, and contexts where conventional sensory channels are burdened or unavailable.

**Fig. 4.**
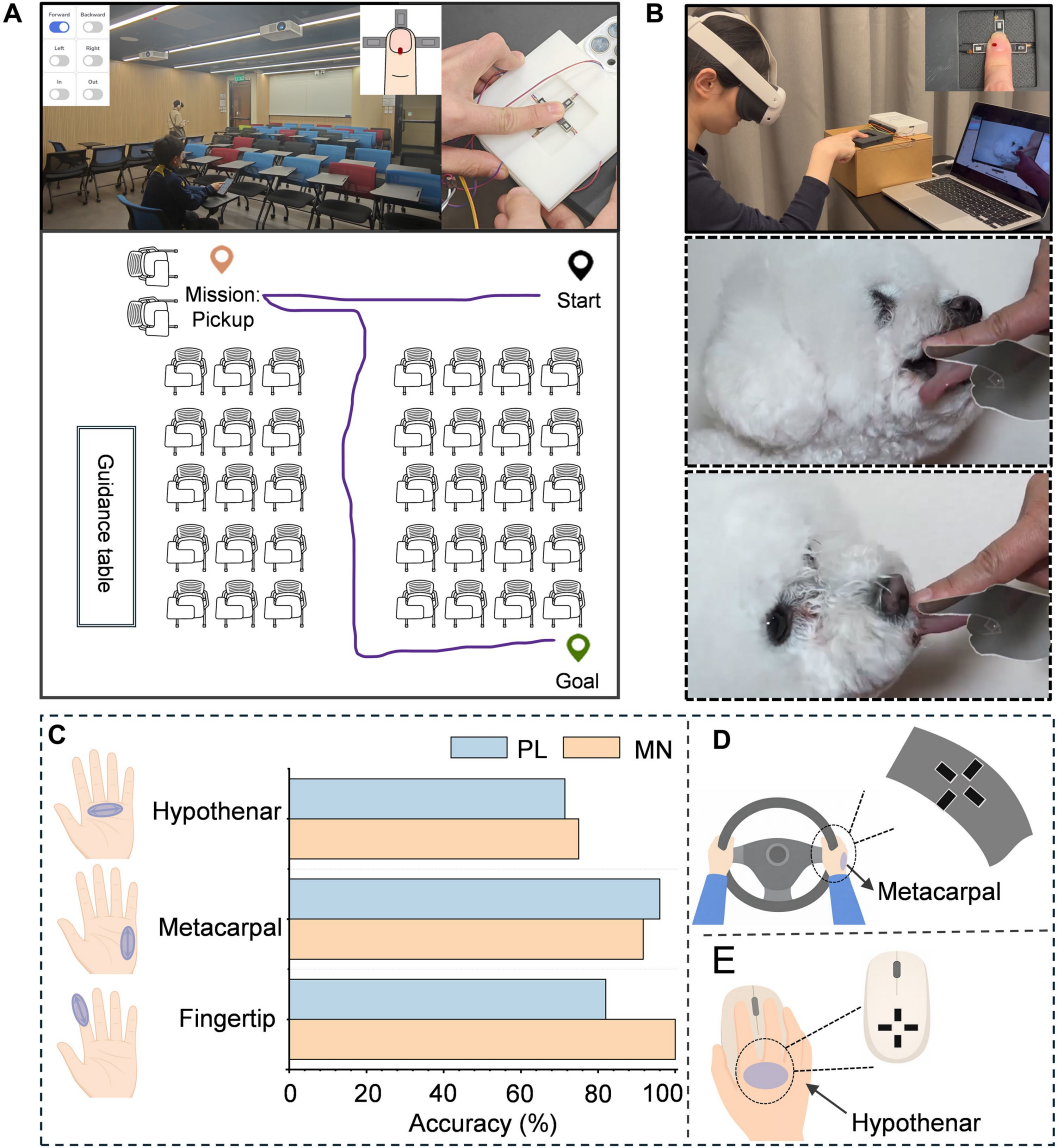
Demonstration of TADT in realistic human-machine interaction scenarios. (**A**) Real-world navigation tasks from tactile cues. (**B**) VR demonstration setup simulating dog licking interaction with dynamic tactile focus control. (**C**) Recognition accuracy comparison across different hand anatomical locations for plane and microtextured interfaces. Potential integration of TADT in hands-busy interaction scenarios: (**D**) steering-wheel control and (**E**) mouse operation.

We also integrated our device with a virtual reality (VR) environment to simulate naturalistic haptic feedback. As shown in Fig. 4B, participants wore a VR headset and touched the surface of the microtextured enhanced actuator array while interacting with a virtual dog. The dog licked the user’s hand from either an upward or lateral direction, with the corresponding tactile focus dynamically modulated using phase or amplitude control (movie S2). Participants reported clear perceptions of the licking direction, suggesting that the dynamic focal rendering was intuitive and immersive. This demonstration highlights the potential of our system for enriching haptic feedback in VR/augmented reality environments.

The effect of actuator placement on perception performance was explored by attaching two actuators to three different body sites rich in mechanoreceptors (*57*, *58*): the fingertip, the metacarpal region, and the hypothenar eminence, which also have distinct functional roles in daily interaction. As shown in Fig. 4C, the microtextured enhanced interface consistently outperformed the plane interface across the hypothenar and fingertip. The fingertip achieved the highest recognition accuracy, followed by the metacarpal and hypothenar areas, reflecting the natural gradient in mechanoreceptor density. These sites correspond to commonly used contact areas in hands-busy contexts: The hypothenar region engages prominently during grasp-based tasks such as steering-wheel control (Fig. 4D), whereas the metacarpal region aligns with palm-down contact during tasks like mouse operation (Fig. 4E). These results suggest that TADT can be deployed for task-specific directional cueing in both static and dynamic scenarios, without interfering with manual manipulation.

## DISCUSSION

This study establishes TADT as a static contact vibrotactile interface capable of delivering programmable focal cues beneath the skin surface. The integration of a microtextured interface with phase- and amplitude-controlled actuation overcomes the mechanical impedance of the stratum corneum, enabling both vertical and lateral transmission to deep mechanoreceptors and generating volumetric foci in regions where Pacinian corpuscles are engaged. Psychophysical testing revealed a ~30% reduction in perceptual thresholds and reliable discrimination of multiaxis cues. When combined with focal amplification effects (~62% voltage reduction), these improvements translated into an estimated ~80% reduction in drive power compared with planar interfaces. Demonstrations of nonvisual trajectory tracing and immersive VR feedback highlighted the potential of TADT as an intuitive communication channel, suggesting broad implications for human-machine interaction, rehabilitation, and immersive media, where hands-free, low-power, high-resolution tactile guidance could complement visual and auditory information.

Although our experiments used linear resonant actuators (LRAs) to showcase TADT, the underlying principles are not specific to this actuator type. ERM motors, piezoelectric actuators, electret-based actuators, and other vibrotactile devices that can generate controllable mechanical vibrations within the sensitivity range of human mechanoreceptors (typically 100 to 300 Hz) are, in principle, compatible with this approach. Because the proposed method enhances energy transfer at the skin interface and relies on phase-amplitude modulation rather than actuator-specific mechanics, it can in principle be adapted to these alternative devices. This suggests that TADT may serve as a generalizable strategy across multiple vibrotactile platforms, provided that future work verifies its performance under different actuation modalities. In our computational model, the multilayer skin was represented using frequency-independent elastic properties. This simplification enabled clear comparison of geometry-dependent coupling trends within a tractable framework but does not fully capture the viscoelastic and dispersive behavior of skin across frequency. Future work incorporating layer-specific viscoelastic constitutive laws will allow a more accurate representation of the biological frequency dependence. At the same time, the present implementation has limitations that temper its immediate applicability. Recognition accuracy, though consistently above chance, remains below levels required for seamless deployment in commercial product, in part due to the relatively large footprint of the actuators and manufacturing errors that could potentially be ameliorate by scaled manufacturing techniques.

## MATERIALS AND METHODS

### Ethics declarations

The study was conducted by the ethical standards set by the Institutional Review Board of The Hong Kong Polytechnical University (HSEARS20250102004) and the Laboratory animal welfare and ethics committee of the Army Medical University (SYXK 2022-0018). All participants were informed of the experimental procedures and provided written informed consent before participation.

### Measurement of individual STs

Each participant’s ST was identified using a three-phase adaptive staircase method. First, the motor threshold was determined as the minimum amplitude that elicited an involuntary thumb twitch. From this reference point, amplitude was decreased in 10% steps until the participant reported perceiving no more than two stimuli out of six repetitions. Then, the amplitude increased by 5% until at least four of six were perceived. Following this, finer adjustments were applied: The amplitude was decreased by 3% and increased again by 1% increments until the participant’s detection rate stabilized at ~50%, which was defined as their ST. Each amplitude was delivered in blocks of six pulses with pseudorandomized interstimulus intervals. Participants were asked to report the number of stimuli they perceived per block, without receiving feedback. Participants completed the procedure for both the plane and microtextured integrated actuator conditions, presented in a randomized order on the same fingertip.

### Subjective robustness evaluation

To examine the robustness of perceptual enhancement by the microtextured interface, 10 participants (average age range of 18 to 37, 5 females and 5 males) were invited to compare the perceived intensity of a microtextured actuator against a plane actuator driven at the same amplitude and frequency. For each trial, participants first touched the plane interface and assigned it a reference intensity of 5 and then rated the perceived intensity of the microtextured interface relative to this reference. Three sets of supplementary experiments were conducted.

For moisture-condition testing, the fingertip was prepared in one of three conditions: water-wetted, alcohol-wiped to simulate a dry condition, or coated with artificial sweat. For contact-pressure testing, participants were instructed to apply either 4- or 8-N normal force with real-time monitoring using a pressure sensor. For participant-group comparison, data were grouped into male and female cohorts to examine whether the observed enhancement remained consistent across participants with potentially different skin properties. All ratings were collected under otherwise identical actuation conditions.

### Relative subjective intensity rating for microtexture height comparison

We performed relative subjective intensity rating to compare the perceptual effect of different interface geometries. Microtextured actuator interfaces with heights of 100, 150, 200, and 250 μm were tested under the same excitation frequency and amplitude. In each comparison, participants first touched the plane actuator interface, which was defined as the reference condition and assigned a fixed score of 5. They then touched the corresponding microtextured actuator and rated its perceived vibration intensity relative to the plane reference. Scores above 5 indicated stronger perceived vibration than the plane interface, a score of 5 indicated similar intensity, and scores below 5 indicated weaker perceived vibration. These ratings were used to generate the data shown in Fig. 2E.

### Animal experiment

In our animal experimental protocol, age-appropriate Sprague-Dawley rats (6 weeks) were anesthetized using isoflurane (3 to 4% induction, 1 to 2% maintenance) before microtexture application. The microtextured device was applied with standardized pressure to three designated sites on the plantar surface of both forepaw and hindpaw footpads in either static mode or vibration mode (300 s). Next, animals were humanely euthanized by cervical dislocation. Full-thickness footpad skin samples were immediately harvested, fixed in 4% neutral buffered polyoxymethylene for 24 hours, processed through standard paraffin embedding protocols, and sectioned at 5-μm thickness. H&E staining was performed on all tissue sections, and specimens were examined under light microscopy at ×40, ×100, ×200, and ×400 magnifications by a histopathologist blinded to experimental groups (fig. S10). Histomorphometry assessment focused on epidermal thickness, stratum corneum integrity, inflammatory cell infiltration, and dermal collagen organization.

### Finite element modeling of vibrotactile coupling in multilayer skin

A harmonic plate load was applied to the top surface of the model, either directly or through a coupled microtextured array interface, to simulate the excitation from a vibrating patch. Two configurations were considered: (i) with a rigid interface directly in contact with skin and (ii) with a microtextured array embedded at the interface. This allowed for directly comparing the wave transmission characteristics under different coupling conditions. In the present implementation, each skin layer was modeled as a linear elastic material with frequency-independent properties. This simplified formulation was adopted to isolate the effect of interface geometry on strain transfer and to enable a direct comparative analysis between plane and microtextured coupling under the same constitutive assumptions. The model was therefore intended as a first-order mechanical approximation for identifying geometry-dependent enhancement trends, rather than as a full viscoelastic representation of biological skin. Incorporating frequency-dependent viscoelastic constitutive laws will be an important direction for future refinement.

Boundary conditions were defined as follows: The bottom surface of the dermis layer was fixed, all side faces of the three skin layers were assigned low-reflecting (absorbing) boundary conditions, and fixed constraints were applied at the four bottom corners to prevent rigid body motion. Frequency-domain simulations were conducted to analyze the spatial distribution of displacement and strain fields within the skin tissue, particularly focusing on the dermis layer.

### Computational framework for microtexture-driven vibration propagation

As shown in Fig. 2F, the displacement field ${\vec{u}}_{\text{ref}}\left(\vec{r}\right)$ generated by a single microtexture placed at the origin is precomputed. This field captures the spatial response of the skin tissue to a unit excitation and serves as the building block for the full-array computation. For any microtexture located at position $\vec{p_{i}}$, its contribution to the displacement field at an arbitrary observation point $\vec{x}$ can be obtained by a coordinate shift

$${\vec{u}}_{i}\left(\vec{x}\right)={\vec{u}}_{\text{ref}}\left(\vec{x}-{\vec{p}}_{i}\right)$$ (1)

This relation allows the reuse of the precomputed reference field to evaluate the response from all needles in the array without computing a complete simulation model that includes the entire array, thereby reducing computational cost. The total displacement field $\vec{U}\left(\vec{x}\right)$ at the observation point due to the entire MN array (with *N* needles) is calculated by summing up the contributions of all individual needles

$$\vec{U}\left(\vec{x}\right)=\sum_{i=1}^{N}{\vec{u}}_{\text{ref}}\left(\vec{x}-{\vec{p}}_{i}\right)$$ (2)

This approach leverages the system’s linearity to efficiently synthesize the complex distribution of fields resulting from a dense microtextured array.

### Device assembly and configuration

The multimodal feedback device was assembled on a custom-designed 3D printed base, which serves as a fixture for precise positioning and stable operation of the actuators. Four LRAs were mounted in pairs on the four vertices of the base, with each opposing pair spaced 1 cm apart to ensure symmetrical force distribution. Each LRA is independently controlled by a dedicated excitation signal to enable programmable and spatially resolved haptic patterns. At the contact interface, each actuator was equipped with a custom-fabricated microtextured patch, designed to enhance tactile perception by modulating the skin-actuator interaction at the microscale. During operation, the user places their finger into a cross-shaped opening reserved at the center of the base, allowing the fingertip to naturally and evenly contact all four actuators simultaneously.

### Subjective experiments for direction recognition

Twenty healthy participants with normal tactile function and no history of neurological disorders were recruited under Institutional Review Board–approved protocols with informed consent. Participants were seated comfortably and asked to identify the direction of vibrational stimuli rendered along six possible axes: upward, downward, leftward, rightward, inward, and outward. Stimuli were delivered in pseudorandomized order using dynamic targeting, with each direction repeated three times per trial block. No visual or auditory cues were provided, and participants were instructed to respond solely on the basis of tactile perception.

### Qualitative subjective evaluation of focal tactile perception

To characterize the perceptual quality of the proposed focal tactile feedback, participants compared the dynamic focal stimulation generated by the device with conventional surface vibration produced by a single actuator. After experiencing both conditions, they rated four descriptors—more continuous, more localized, sharper, and deeper—using a five-point agreement scale, where 1 indicated strong disagreement and 5 indicated strong agreement that the focal feedback exhibited the corresponding sensation more strongly than the conventional surface vibration. These ratings were used to generate Fig. 3J.
