# Supplementary material for: Transforming static interfaces into tactile channels with steerable transdermal foci
Source: Sci Adv. 2026 Jul 8;12(28):eaef2372. doi: 10.1126/sciadv.aef2372 (PMC13344323; doi:10.1126/sciadv.aef2372)
Supplement: Supplementary file 1 — Figs. S1 to S15 Legends for movies S1 to S3 [file sciadv.aef2372_sm.pdf]

Science Advances
AAAS

Supplementary Materials for

Transforming static interfaces into tactile channels with steerable transdermal foci

Qiutong Liu *et al.*

Corresponding author: Yi Tang, tang-yi.tang@polyu.edu.hk; Jiezhou Pan, panjz@tmmu.edu.cn; Yuan Ma, y.ma@polyu.edu.hk

Sci. Adv. **12**, eaef2372 (2026)
DOI: 10.1126/sciadv.aef2372

The PDF file includes:

Figs. S1 to S15
Legends for movies S1 to S3

Other Supplementary Material for this manuscript includes the following:

Movies S1 to S3

Fig. S1. SEM images of the microneedle array interface and a geometric schematic with labeled dimensions.

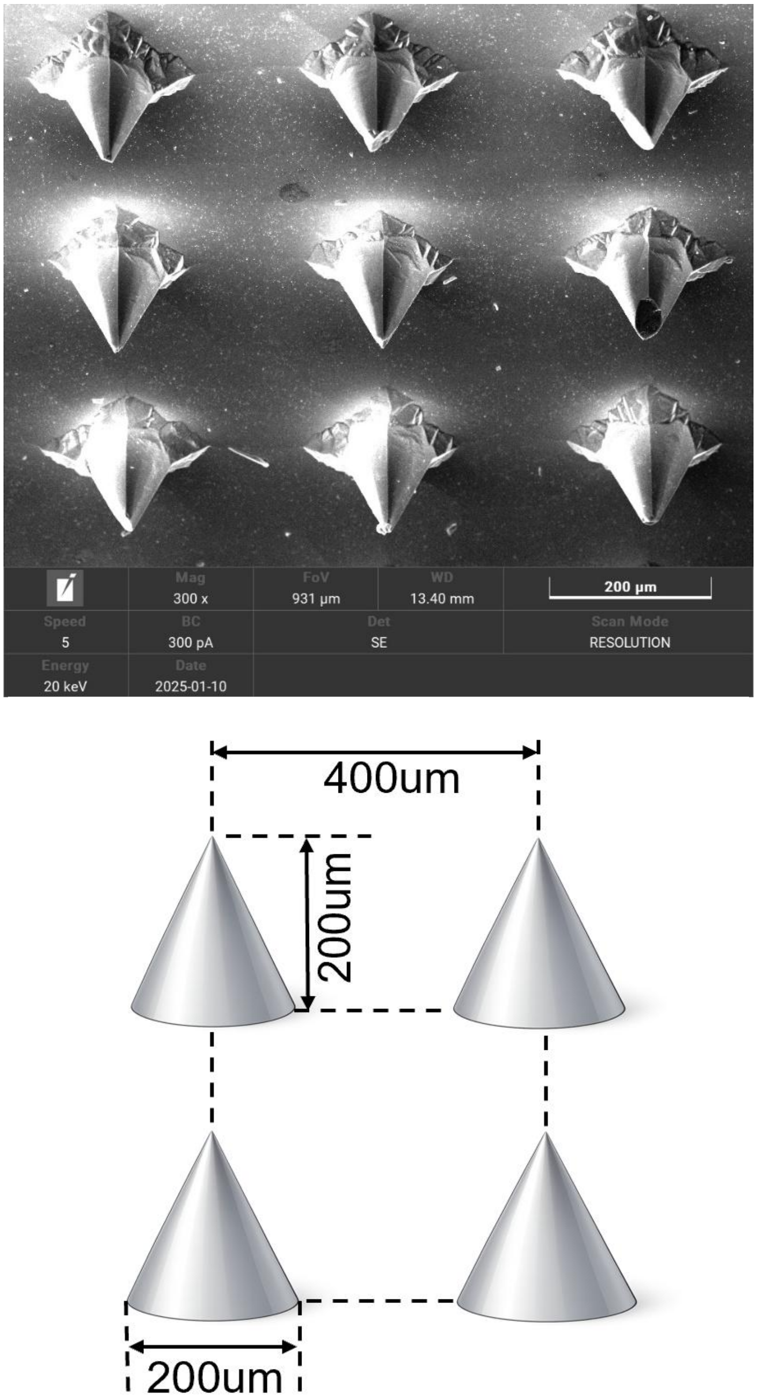

Fig. S2. Displacement amplitudes at 200 µm depths during Y-axis vibration for actuators with and without microneedles.

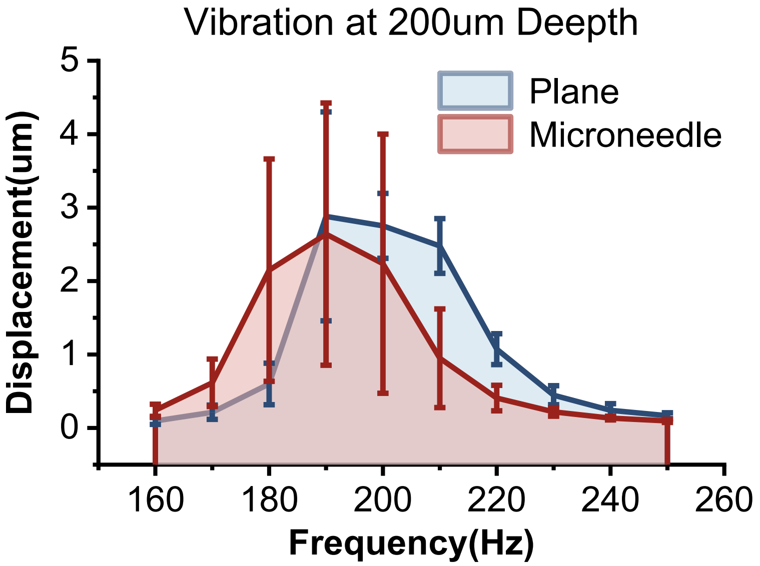

Fig.S3. Perceptual robustness of the micro-textured interface across fingertip moisture conditions. Participants first touched the plane actuator interface and assigned it a reference intensity of 5, then rated the perceived intensity of the micro-textured actuator under the same driving condition. Three fingertip conditions were tested: water-wetted, alcohol-wiped to simulate a dry condition, and artificial sweat applied to the fingertip. In all cases, the micro-textured interface was perceived as stronger than the plane reference.

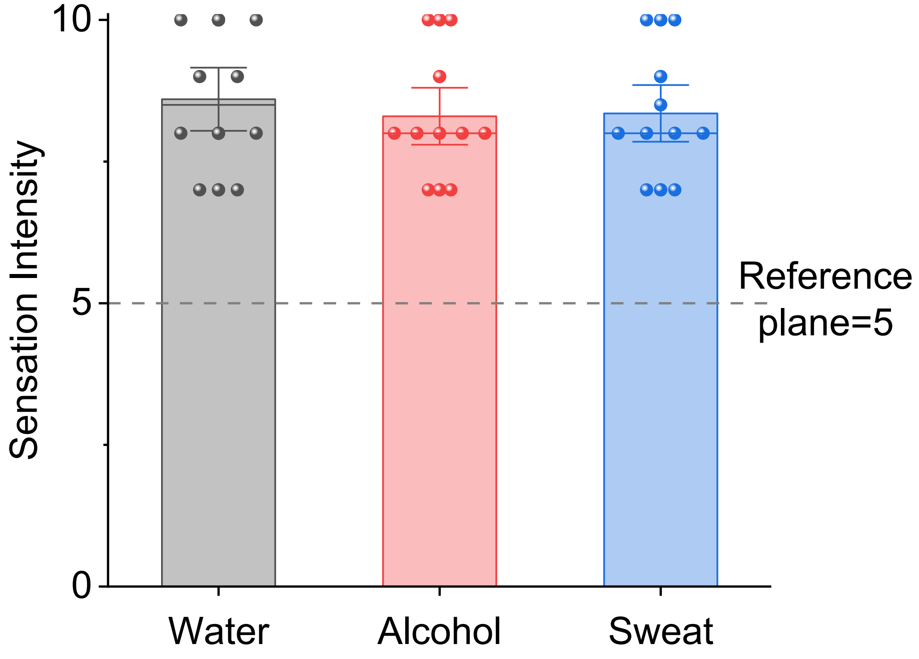

Fig.S4. Effect of contact pressure on subjective intensity enhancement of the micro-textured interface. Participants compared the perceived intensity of the micro-textured interface with a plane reference (intensity = 5) under controlled normal forces of 4 N and 8 N. The micro-textured interface remained perceptually stronger at both pressures, although the relative enhancement was moderately reduced at the higher preload.

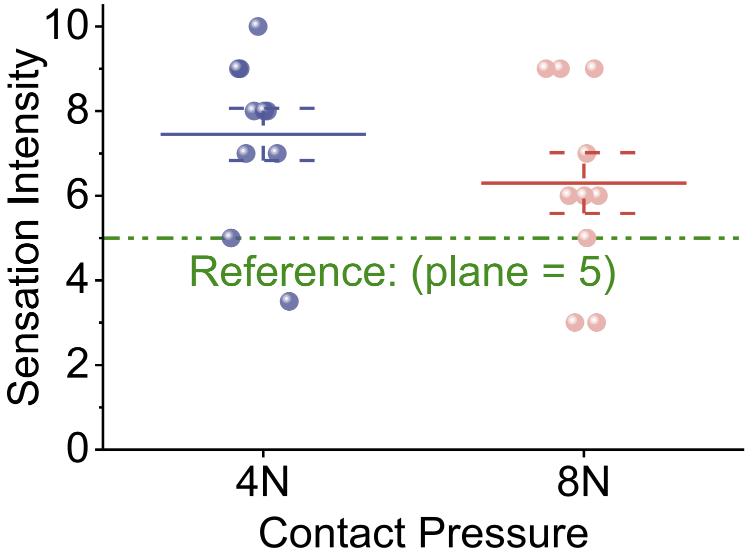

Fig.S5. Perceptual enhancement across male and female participant groups. Subjective intensity ratings of the micro-textured interface relative to a plane reference (intensity = 5) for male and female participant groups. Both groups perceived stronger stimulation from the micro-textured interface, indicating that the enhancement is observable across participant groups with potentially different skin properties.

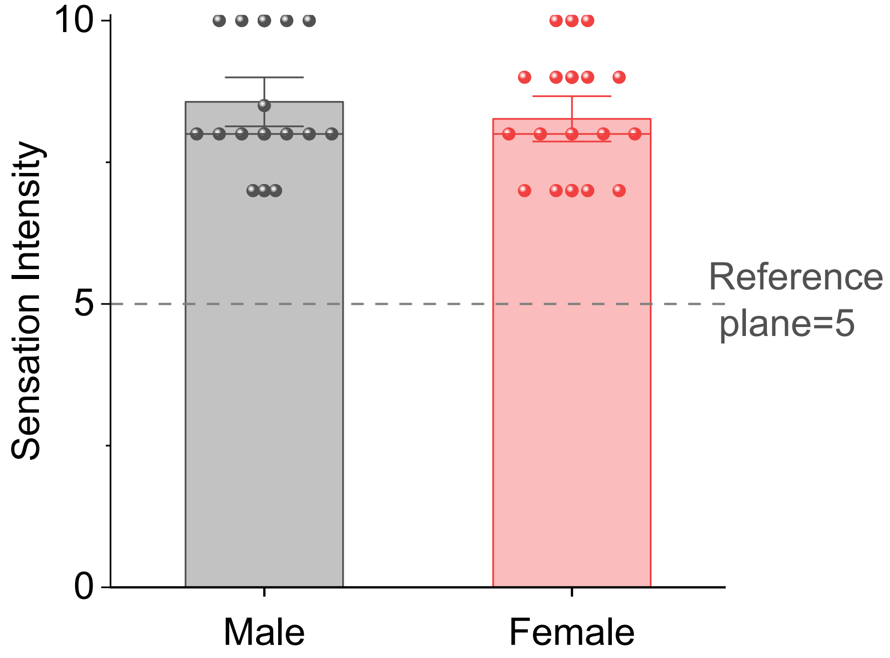

Fig. S6. Frequency-dependent subjective intensity comparison between plane and micro-textured interfaces. Participants first touched the plane actuator and assigned it a reference intensity of 5, and then rated the perceived intensity of the micro-textured actuator under the same driving condition. Two excitation frequencies, 50 Hz and 100 Hz, were tested. The micro-textured interface produced higher perceived intensity at 100 Hz, whereas at 50 Hz its rating was lower than that of the plane reference, indicating a frequency-dependent perceptual benefit.

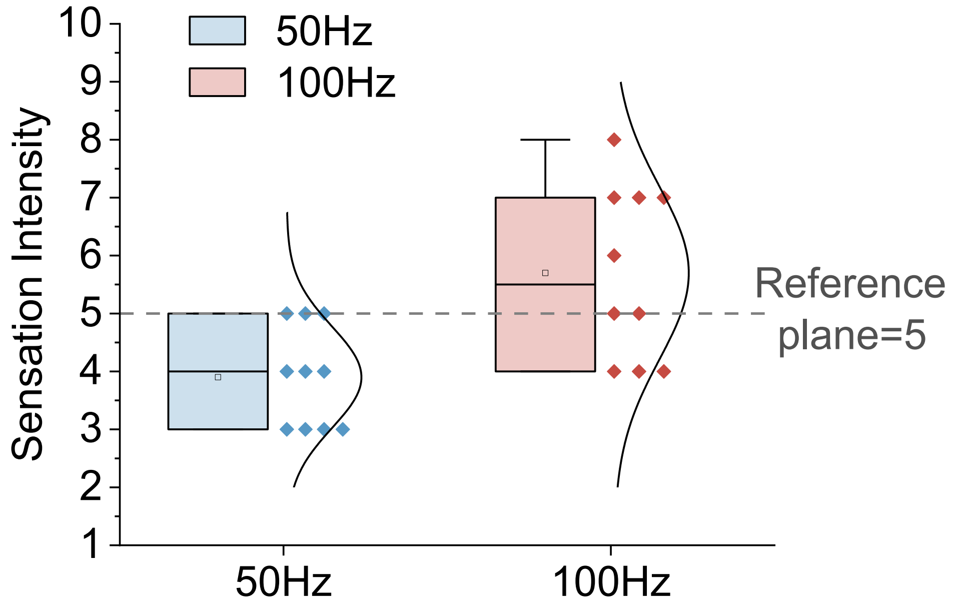

Fig. S7. Effect of micro-texture shape on simulated dermal coupling.
Simulated comparison of three representative micro-texture geometries, including a sharp cone, a rounded-corner cone, and a rectangular protrusion. The bar plot summarizes the displacement measured at the dermal layer, and the corresponding strain/displacement fields are shown on the right. Under the idealized simulation conditions used here, all three geometries enhanced subsurface mechanical transmission to a similar extent, with the rectangular protrusion showing a slightly higher value.

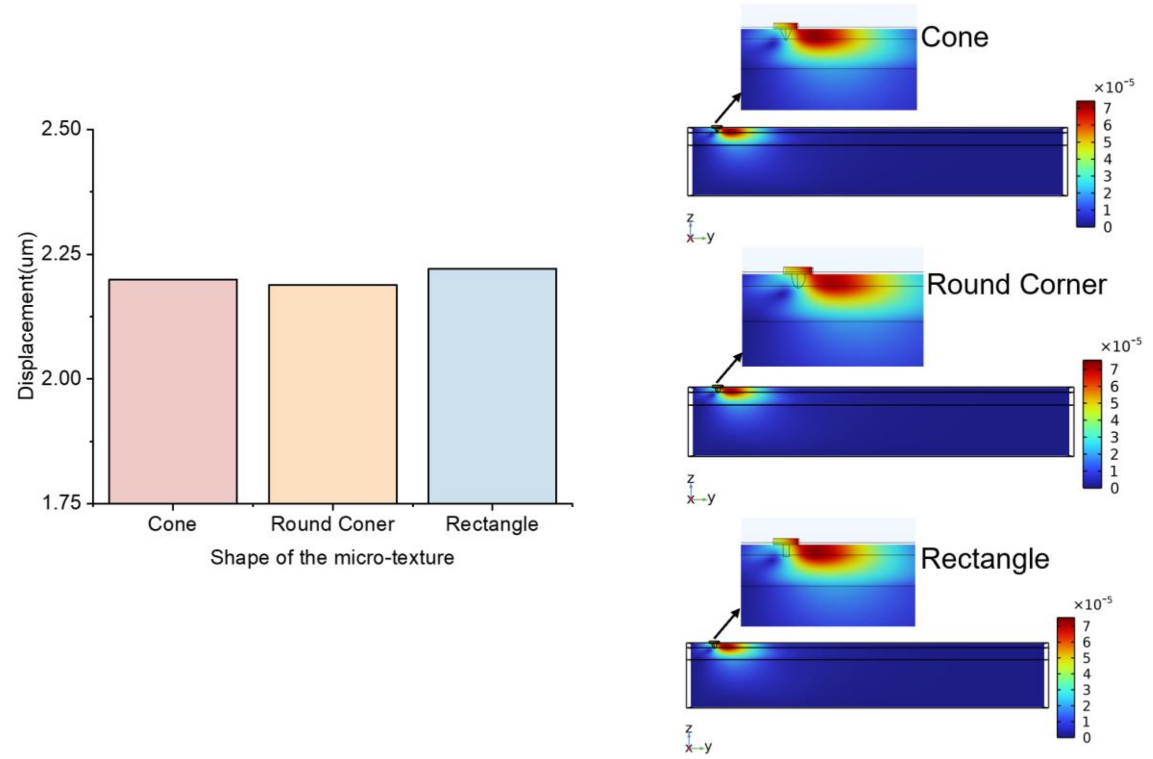

Fig. S8. Simulated Time-Domain Displacement at 7 mm for Plane and Microneedle Interfaces.

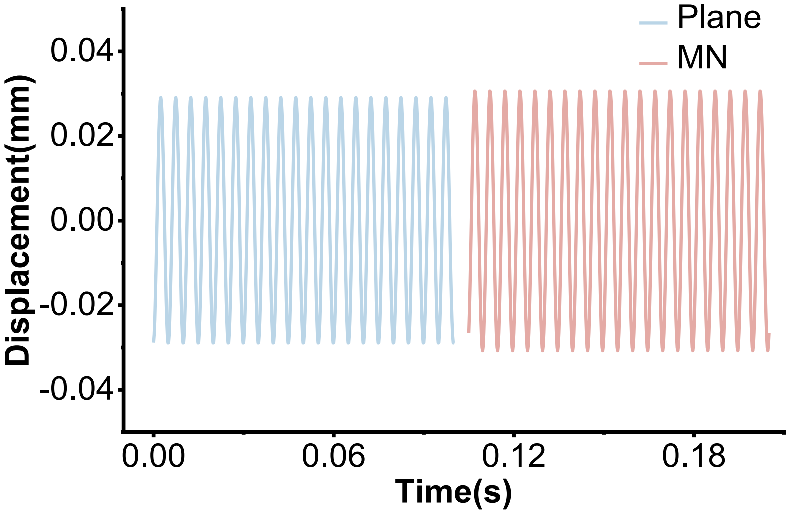

Fig. S9. Displacement at 5 mm under different microneedle array layouts.

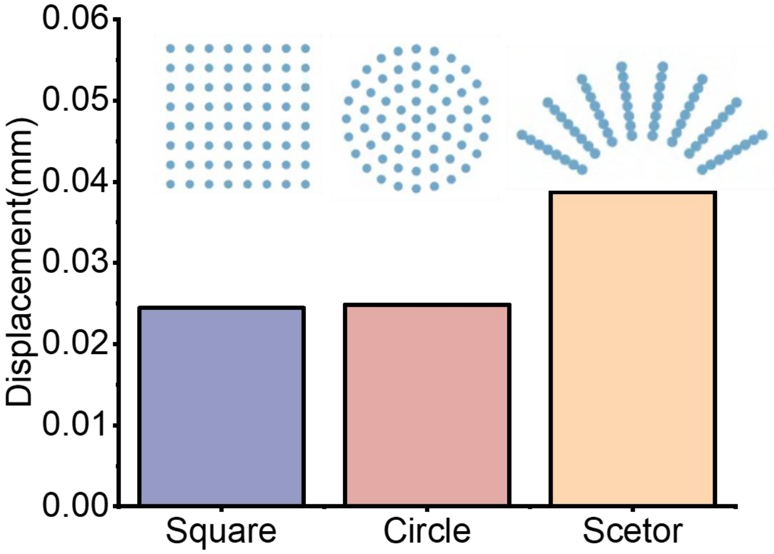

Fig. S10. Histological evaluation of microneedle application on SD rat footpads. (**A**) Schematic and representative photographs of the animal experiment, showing the tested paw regions and the application of the micro-textured interface at the designated contact sites. Sites **a-c** indicate the representative forepaw and hind paw regions used for the histology evaluation. In panel **A**, the leftmost schematic was assembled using graphics created in BioRender. Pan, P.(2026) https://BioRender.com/f6u2vow; all other elements were created by the authors. (**B**) Representative photomicrograph of H&E-stained tissue section from control group footpad exhibiting normal epidermal and dermal architecture. (**C**) Representative photomicrographs of H&E-stained tissue sections following microneedle application in static mode and (**D**) vibration mode, demonstrating visible tissue depression without disruption of stratum corneum integrity. In (**C**) and (**D**), panels a-c corresponds to different indentation locations, and the dashed boxes indicate regions selected for higher-magnification analysis. Panels I-III show magnified views of the boxed regions in panels a-c, respectively. Panels S11Ba, S11CI, S11DI are reproduced from Fig. 2H-J (main text) for clarity, respectively.

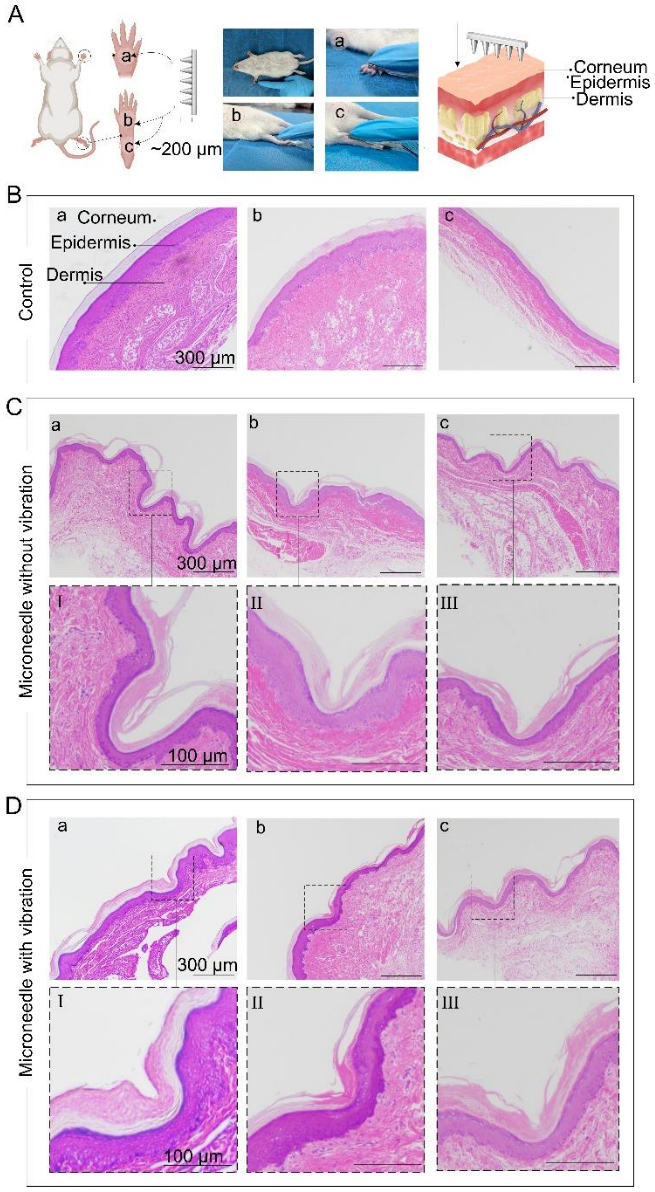

Fig. S11. In-plane vibration localization at Positions 1, 3, and 5 in the 600 µm depth region. Time-domain lateral vibration signals measured at Positions 1, 3, and 5 within the 600 µm depth plane.

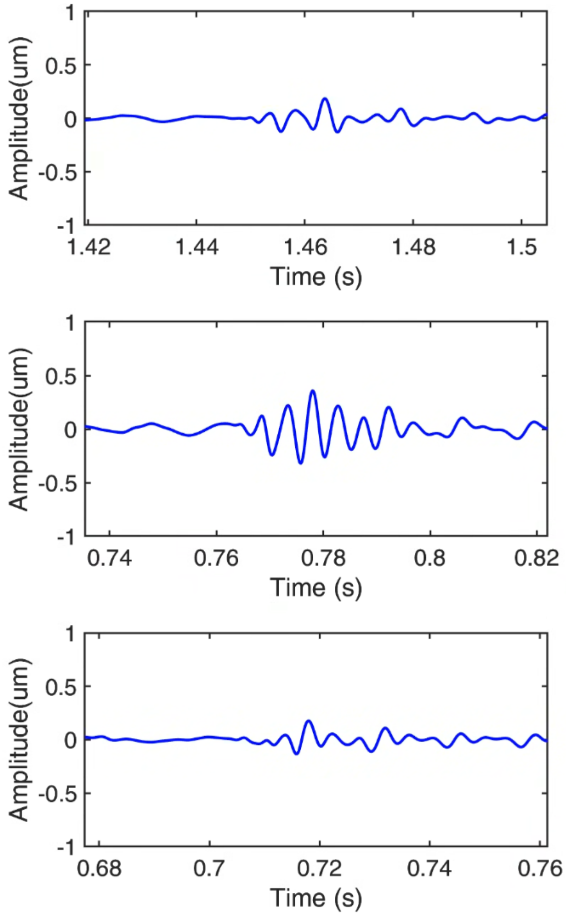

Fig. S12 Actuator arrangement and driving signals for directional tactile stimulation. (**A**) Schematic of the four-actuator configuration surrounding the fingertip. (**B**) Example driving signals used to generate stimulation along the Y direction. (**C**) Example driving signals used to generate stimulation along the X direction. Panel A is reproduced from Fig. 3D (main text).

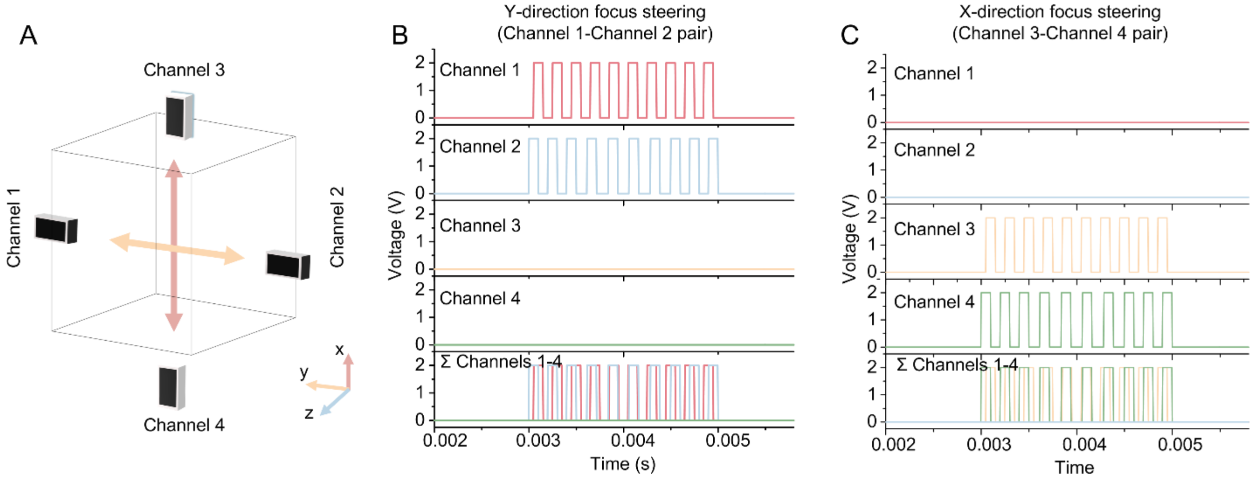

Fig. S13. Actuator configuration and driving signals for vertical tactile stimulation.
(**A**)Schematic illustration of the four-actuator arrangement surrounding the fingertip.
(**B**)Driving signals used to generate stimulation along the Z direction. Panel A is reproduced from Fig. 3E (main text).

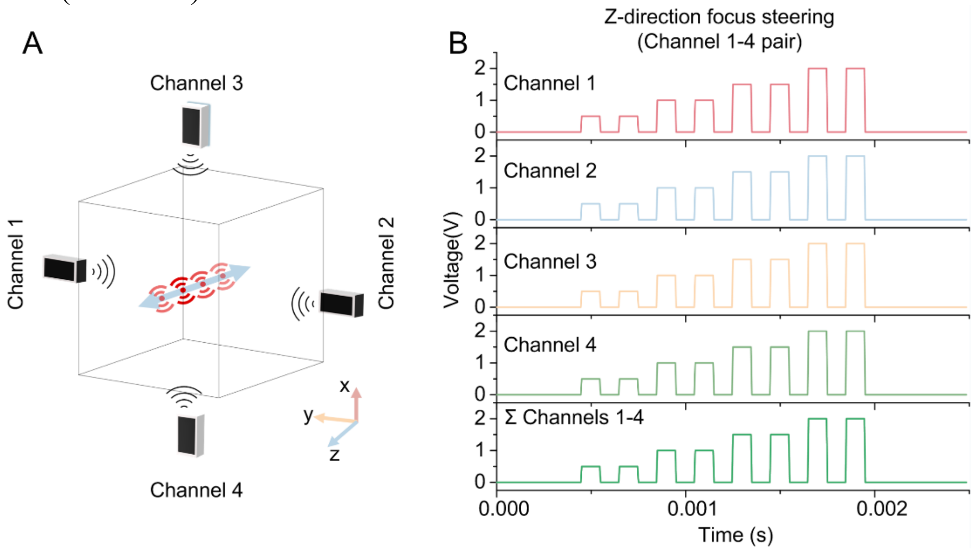

Fig. S14. Direction recognition accuracy with non-coplanar actuator arrangements. Four actuators were mounted on convex and concave curved bases, respectively, and participants performed the same directional recognition task as in the planar configuration. The recognition accuracy remained high in both cases, reaching 90% for the convex arrangement and 80% for the concave arrangement, indicating that TADT remains effective under non-coplanar actuator layouts, although surface curvature influences perceptual performance.

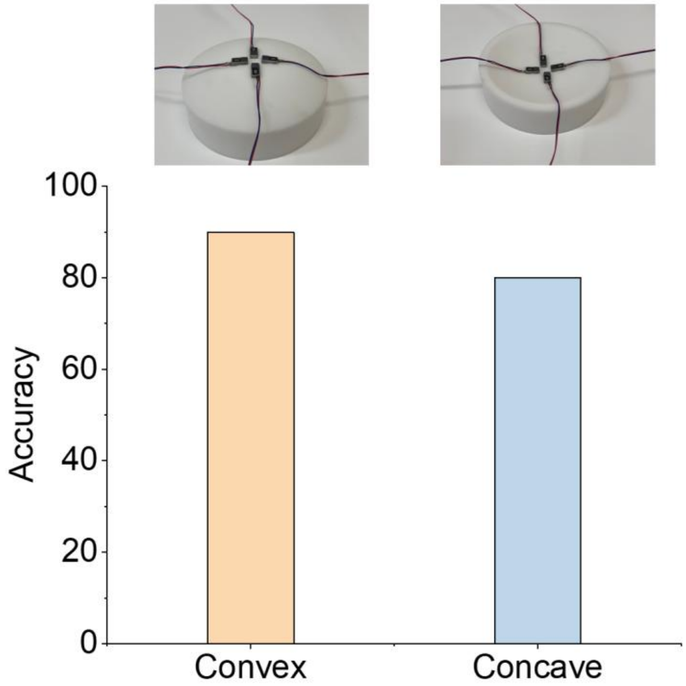

Fig. S15. Detailed assembly of the tactile guidance device for the navigation experiment. The device includes a power module, an MCU-based control board, an analog signal output module, and four actuators.

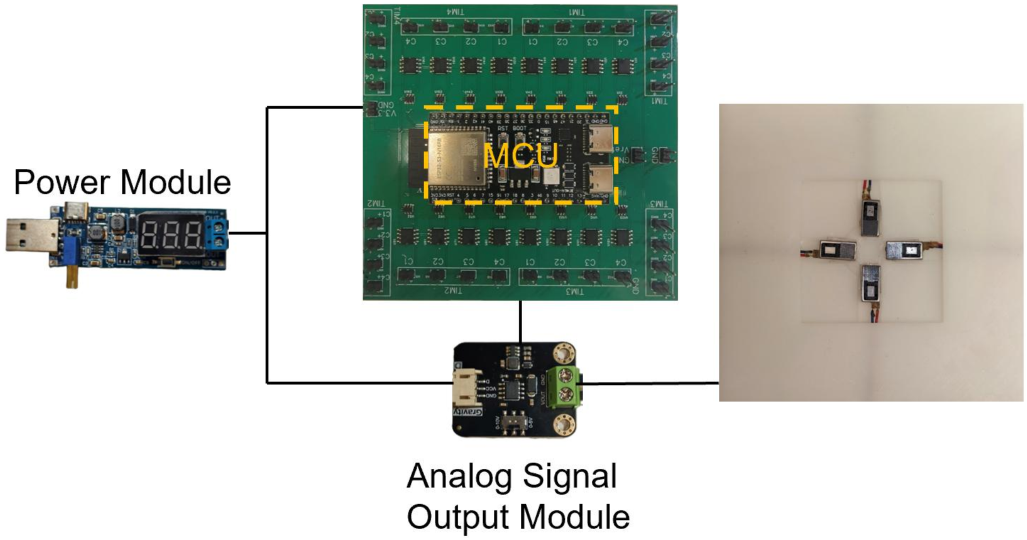

Movie S1 Real-world indoor navigation guided by programmable vibrotactile cues.
Movie S2 Directional tactile rendering in a virtual dog-licking interaction.
Movie S3 Driving signals for X- or Y-direction focus steering via phase modulation between actuators.
